# Dental Caries, Hygienically Considered

**Published:** 1867-01

**Authors:** J. P. H. Brown

**Affiliations:** Augusta, GA.


### DENTAL CARIES, HYGIENICALLY CONSIDERED.

BY DR. J. P. H. BROWN, AUGUSTA, GA.

The treatment of dental caries is unquestionably the pivotal point of Dental Science. To arrest the ravages of decay has always engrossed the attention of the best minds in the profession. And if we consider the subject in a mechanical light, great results have been achieved. Fillings are now inserted which are, for impermeability, solidity and finish, everything that can be desired. And the file, though often abused, has been, no doubt, used judiciously and effectively. To say that such operations upon the teeth do not check and arrest decay, is to make the assertion of an ignoramus.

All these mechanical processes are well and proper, as far as they go, in retarding the progress of decay. The only fault to find is, that they grapple with the effects, to the neglect of a due consideration of the causes of caries. But can any one say that these causes are clearly determined? If the pathology of decay be clearly understood, then why is it that we have so many theories advanced for its solution? Thus we have one hypothesis that makes it no disease at all, but purely the result of chemical decomposition, and dependent upon the operation of chemical laws for its origin and progress; while another makes it a *chemico-vital* process. One English writer, Mr. Bridgeman, has advanced a very ingenious theory, showing that it is the result of "electro-voltaic" action going on within the tissues of the tooth; while Mr. C. Spence Bate contends that the active agent of solution is carbonic acid generated in immediate contact with the salts of the tooth which it dissolves. These hypotheses are maintained by men equally eminent in the profession, and equally conversant with the true histological character of the Dental tissues.

It is not my purpose in the present article to take sides or issue with any of the above theories; nor do I wish to advance any pet notion of my own, nor do I expect to put forth anything which is not already known; but only to offer a few thoughts which may possibly lead others to observe, investigate and note. Our great specialty of Dentistry being still in its infancy, the most of us are yet students, though we have years of experience.

Aetiologically speaking, the causes of decay may be divided into two kinds, viz.: exciting, and predisposing or constitutional. That the first are pretty well understood may be attested by the practice of every faithful operator But in regard to the second, it is very evident that their pathology is not so clear; and to these I purpose confining myself in the present paper.

What I understand by predisposing or constitutional causes of caries are those defects in the structure of the Dental tissues, which seem to be a want of a proper proportion between the animal and earthy elements; whereby the tooth has but little power to resist the action of those chemical agents which come in contact with it. And, furthermore, as the weak *physique* of these tissues is the result of malassimilation, a careful study of those influences and conditions which impair this function is of the greatest importance.

Defective power to assimilate healthy tooth-structure is as transmissible from parent to child as any other weak organ or faculty. This can be seen every day in any ordinary practice. Children are often found with teeth resembling some parent, not only in shape, but also in the locality and peculiarity of the decay. The writer has met with a case where the right lateral incisor was wanting, and the same tooth was not only deficient in the mother but also in the maternal grandfather. He also knows of a family whose several members never had any teeth, and the edentulous jaw can be traced back through several generations. Dr. Fitch, in his *System of Dental Surgery*, relates a similar case.

Disease of nearly every kind in the parent, operates to produce in the child mal-assimilation of the elements of the dental tissues, and consequently constitutional degeneracy of the dental organs. Dyspepsia, rheumatism, and particularly the *exanthematous* and *impetiginous* diseases seem to be the most active in disordering this function. Of the latter type I regard syphilis as prominent in stamping its baneful effects upon the teeth. It has been shown by Mr. Hutchinson, and other pathological observers, that this insidious disease has the power of smouldering in the system from generation to generation, and giving to the teeth a peculiar formation as well as a vitiated texture. This writer remarks: "Although the temporary teeth often, indeed usually, present some peculiarities in syphilitic children, of which a trained observer may avail himself, yet they show nothing which I dare describe as worthy of general reliance. The *central upper incisors of the second set are the test teeth*, and the surgeon not thoroughly conversant with the various and very common forms of dental mal-formation will avoid much risk of error if he restricts his attention to this pair. In syphilitic patients these teeth are usually short and narrow, with a broad vertical notch in their edges, and the corners rounded off. Horizontal notches or furrows are often seen, but they, as a rule, have nothing to do with syphilis. If the question be put, are teeth of the type described pathognomonic of hereditary taint? I answer unreservedly, that when well characterized, I believe they are. I have met with many cases in which the type in question was so slightly marked that it served only to suggest suspicion, and by no means to remove doubt, but I have never seen it well characterized without having reason to believe that the inference to which it pointed was well founded."*

The teeth are governed, in proportion to their vitality, by

* A Clinical Memoir on Certain Diseases of the Eye and Ear, consequent on Inherited Syphilis. London. 1863.

the laws of disintegration and reparation, like any other portion of the system. This explains why the teeth of many females, after marriage, decay so rapidly. They enter the marriage relations with so little life-power that, when they become pregnant, they have not sufficient vitality to appropriate to the tooth-germs of the fœtus enough of the inorganic elements from the food to form healthy dental tissue. In such cases the demands of the fœtus upon the mother for the phosphate of lime, leaving her osseous system in a weak condition. The teeth also furnish their quota of lime, but as their power of reparation is very feeble in comparison to the more vitalized structure of bone, they are left constitutionally depraved, and illy prepared to resist the chemical action of the vitiated secretions of the mouth. Hence it is that the offspring of such mothers have teeth of so frail a quality, that the best and most skillful operations upon them can prove of only temporary benefit.

In the treatment of such cases I have more faith in a correct observance of the hygienic agents, and in the application of those means tending to improve and invigorate the general system, than in the administration of such remedies as phosphate of lime. It is the opinion of the writer that the trouble is not so much owing to a deficiency of the lime salts in the food, as it is to a *want of sufficient power, in the mother or child, to assimilate the necessary constituents of a well organized tooth.*

There can be no doubt that our food, as it is now prepared by what is termed the *science* of modern cookery, is not only poorly fitted to keep our organs of digestion in a healthy and normal condition, but it is also inadequate to supply our teeth with that exercise in mastication which is indispensable to their health. Teeth on the side of the mouth that is not used, or those that have lost their antagonists, soon become foul, decayed, and diseased in their sockets. Whereas we often find decay arrested by exercise, as for instance, in a broad but shallow cavity on the grinding surface of a molar.

And by those inexplicable and wonderful laws of hereditary descent, the impaired constitution of these non-exercised teeth, in the mouth of the parent, is liable to be impressed upon the denture of the offspring.

The influence of plain and coarse diet, and out-door exercise, in promoting healthy organs of mastication, can be seen in the negroes in the South, by comparing the sound teeth of field hands with the decayed teeth of house-servants, who eat of the same food that the whites do. The same can be seen in the peasantry of Europe. They eat plain and wholesome food, take plenty of exercise, and generally have sound teeth well arranged in finely developed arches. But when you get into the large cities where the living is more artificial, and the food highly seasoned and stimulating, the teeth are as badly decayed as they are in our own country.

